# Annotated genome and transcriptome of the endangered Caribbean mountainous star coral (*Orbicella faveolata*) using PacBio long-read sequencing

**DOI:** 10.1186/s12864-024-10092-w

**Published:** 2024-02-29

**Authors:** Benjamin D. Young, Olivia M. Williamson, Nicholas S. Kron, Natalia Andrade Rodriguez, Lys M. Isma, Nicholas J. MacKnight, Erinn M. Muller, Stephanie M. Rosales, Stephanie M. Sirotzke, Nikki Traylor-Knowles, Sara D. Williams, Michael S. Studivan

**Affiliations:** 1 Cooperative Institute of Marine and Atmospheric Science, Rosenstiel School of Marine, Atmospheric, and Earth Science, University of Miami, Miami, FL USA; 2 Atlantic Oceanographic and Meteorological Laboratory, National Oceanic and Atmospheric Administration, Miami, FL USA; 3 Department of Marine Biology and Ecology, Rosenstiel School of Marine, Atmospheric, and Earth Science, University of Miami, Miami, FL USA; 4 Mote Marine Laboratory, Sarasota, FL USA

**Keywords:** De-novo genome assembly, *Orbicella faveolata*, PacBio circular consensus sequencing, HiFi reads, Long-read sequencing, ISO-seq, Stony coral Scleractinia

## Abstract

Long-read sequencing is revolutionizing *de-novo* genome assemblies, with continued advancements making it more readily available for previously understudied, non-model organisms. Stony corals are one such example, with long-read *de-novo* genome assemblies now starting to be publicly available, opening the door for a wide array of ‘omics-based research. Here we present a new *de-novo* genome assembly for the endangered Caribbean star coral, *Orbicella faveolata*, using PacBio circular consensus reads. Our genome assembly improved the contiguity (51 versus 1,933 contigs) and complete and single copy BUSCO orthologs (93.6% versus 85.3%, database metazoa_odb10), compared to the currently available reference genome generated using short-read methodologies. Our new *de-novo* assembled genome also showed comparable quality metrics to other coral long-read genomes. Telomeric repeat analysis identified putative chromosomes in our scaffolded assembly, with these repeats at either one, or both ends, of scaffolded contigs. We identified 32,172 protein coding genes in our assembly through use of long-read RNA sequencing (ISO-seq) of additional *O. faveolata* fragments exposed to a range of abiotic and biotic treatments, and publicly available short-read RNA-seq data. With anthropogenic influences heavily affecting *O. faveolata*, as well as it*s* increasing incorporation into reef restoration activities, this updated genome resource can be used for population genomics and other ‘omics analyses to aid in the conservation of this species.

**Supplementary Information:**

The online version contains supplementary material available at 10.1186/s12864-024-10092-w.

## Background

Advances in sequencing technologies are providing new opportunities in genome assembly and research, specifically, long-read sequencing methodologies such as PacBio and Oxford Nanopore. Longer stretches of DNA can reduce the number of contigs and improve the classification of highly repetitive regions such as telomeric and centromeric repeats [1–4] which is commonly a problem and more difficult with short-read sequencing. In addition, reductions in the cost have made long-read sequencing methodologies highly accessible and attainable for use in non-model organisms, facilitating new research into inter/intra-population variation, as well as investigations into areas such as gene function and gene coding sequences.

**Fig. 1:**
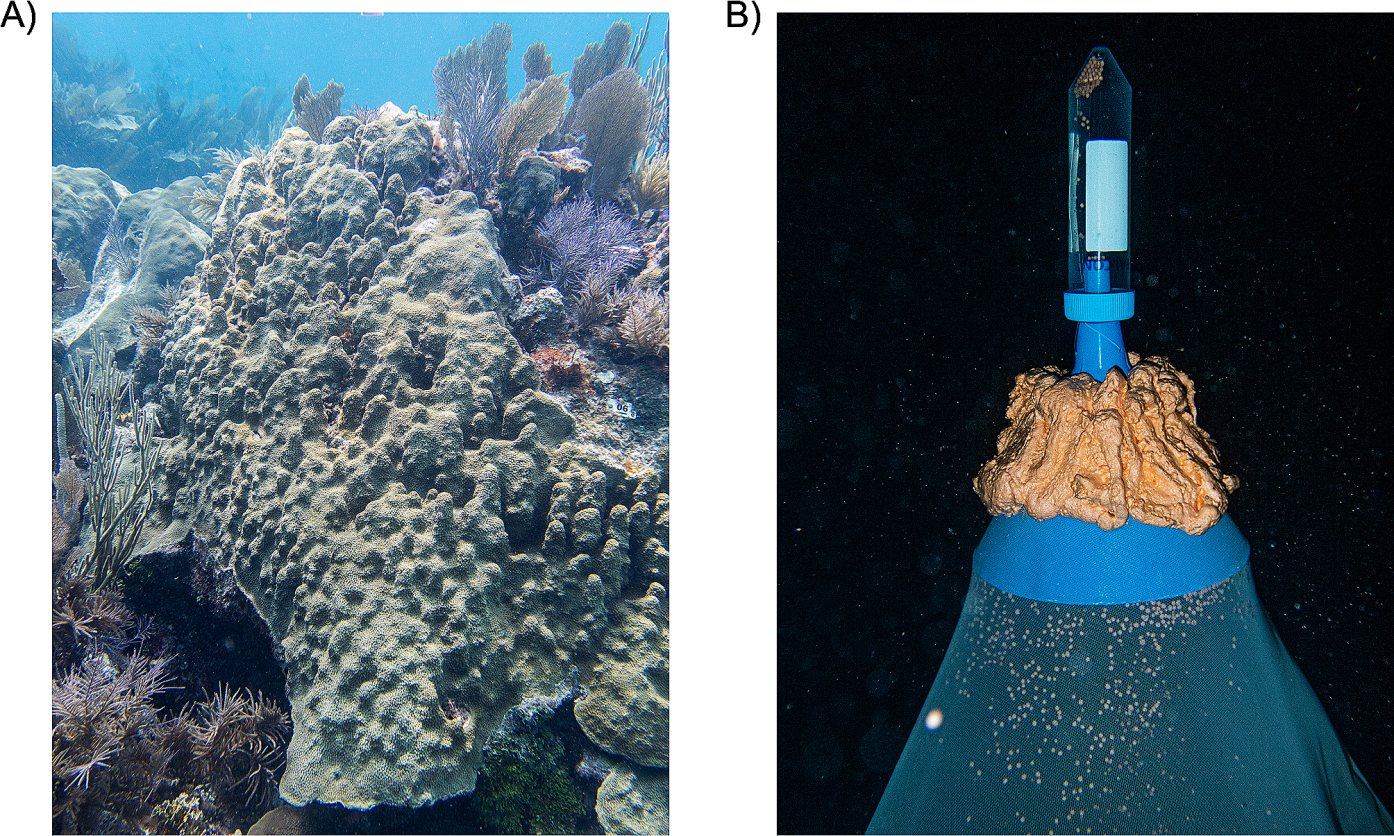
*Orbicella faveolata* on a reefscape and gamete bundle collection methodology. *A*. Picture of an adult *Orbicella faveolata* colony at Horseshoe reef during the day. *B*. Gamete bundle collection methodology apparatus. Picture shows the top of the net which is placed over the adult coral colony, with an attached 50 ml conical centrifuge tube allowing collection of gamete bundles

Stony corals (order Scleractinia) are keystone organisms, providing the framework for subtropical and tropical coral reef ecosystems. At present, the 10 long-read coral genomes that are publicly available all represent those of Pacific coral species [5–10], with no long-read genomic resources available for Caribbean corals. *Orbicella faveolata* (Fig. 1A) is an important reef-building coral in the family Merulinidae in the Caribbean. While historically inhabiting back and fore reefs at a range of depths throughout the Caribbean [11, 12], it is now listed as “threatened” under the US Endangered Species Act [13] and “endangered” on the IUCN red list [14]. Despite ongoing protection efforts, populations of *O. faveolata* have continued to decrease in the Caribbean due to bleaching [15, 16] and disease events, specifically stony coral tissue loss disease [17, 18]. As a result, Caribbean reef restoration activities are currently incorporating *O. faveolata* [19–21]. A highly contiguous and complete reference genome would be an invaluable resource to support restoration efforts through use in ‘omics analysis such as population genetic studies [16, 22] and transcriptomics [23–25]. With local and global anthropogenic influences having drastic effects on coral assemblages and populations [26–29], a well annotated and contiguous genome can also lay the foundations for studies aiming to identify resistance biomarkers within and between populations [30] to variables such as heat stress, disease, and ocean acidification.

Previously, an *O. faveolata* genome was assembled using short-read technology (NCBI accession GCA_002042975.1) [31]. Here, we used long-read PacBio circular consensus sequencing (CCS) of high molecular weight (HMW) DNA extracted from *O. faveolata* sperm collected in the field (Fig. 1B) to assemble a more contiguous and complete *de-novo* genome assembly of *O. faveolata*. We achieved a highly contiguous and complete *de-novo* genome assembly, with long-read RNA-seq (ISO-seq) resulting in better gene prediction. We then discuss how to further improve our resource using approaches such as optical mapping or Hi-C sequencing, and provide applications for the implementation of this genetic resource into ongoing conservation and restoration initiatives.

## Results

### De-novo genome assembly

**Fig. 2:**
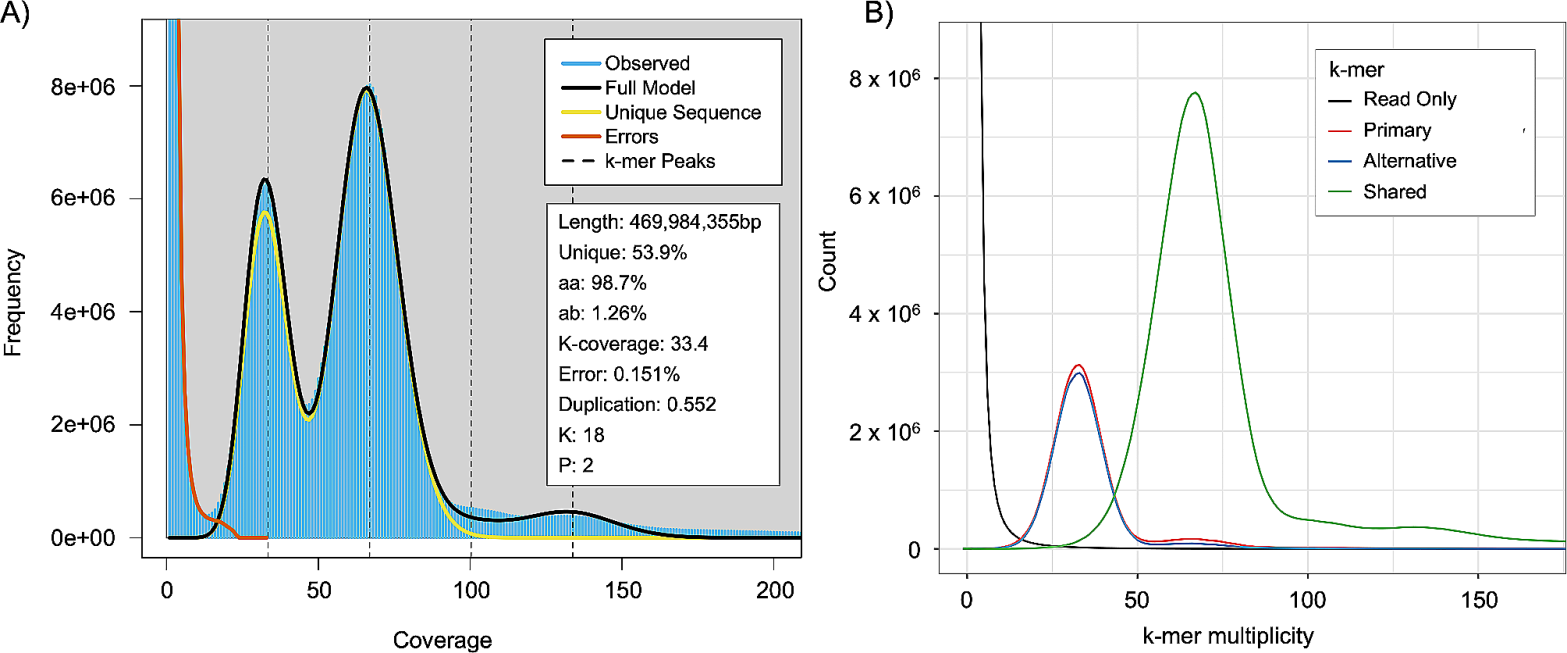
GenomeScope2 and Merqury analyses of the cleaned raw HiFi reads, primary, and alternative assemblies using HiFiasm. *A*. GenomeScope2 linear *k*-mer distributions for *O. faveolata* generated from Merqury output. Black line shows a theoretical diploid model for this species, with computed homozygosity (aa) and heterozygosity (ab) of 98.7% and 1.2% respectively. *B*. Merqury plot of the primary and alternative assemblies using HiFiASM in --primary mode. Default parameters showed duplicates were purged adequately (red and blue lines) resulting in no additional filtering before scaffolding, downstream gene prediction, and annotation steps

High molecular weight (HMW) DNA extracted from *Orbicella faveolata* sperm yielded 2,604,886 HiFi reads (average length = 12,688 base paris (bp), total length = 32,999,915,949 bp). A BLASTn [32] search of the raw HiFi reads identified 54,828 reads which were considered as contaminants (prokaryotic, viral, and UniVec databases) with a bit score > 1000 (2.1% of the raw HiFi reads). After removal of these sequences, 2,550,058 contaminant free (CF) HiFi reads (average length = 12,671 bp, total length = 32,313,799,715 bp) remained and were used for *de-novo* genome assembly. The CF HiFi reads had an estimated sequencing coverage of 99x, a predicted genome length of 469,984,355 bp, ploidy of two, homozygosity of 98.7%, heterozygosity of 1.26%, and duplication of 0.552 (Fig. 2A).

**Fig. 3:**
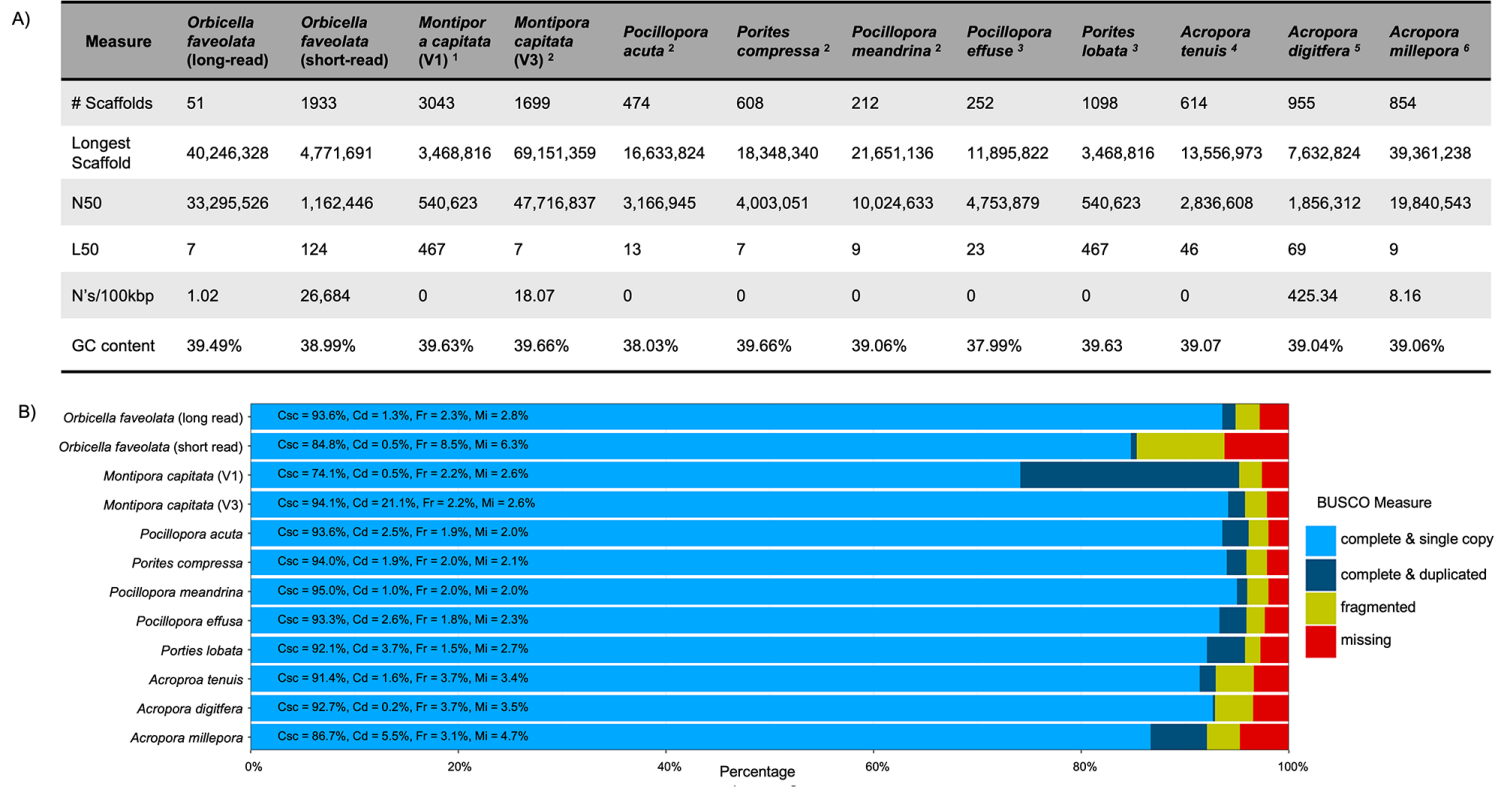
Quast and BUSCO analysis results of long-read stony coral genomes. *A*. Results from Quast analysis of our *de-novo* assembly, previous short read *Orbicella faveolata* assembly, and all publicly available long-read stony coral genomes. *B*. Results of BUSCO analysis using our *O. faveolata de-novo* assembly, the previous short read *O. faveolata* assembly, and all publicly available long-read stony coral genomes with the metazoa_odb10 database. Completeness is split into single copy (light blue) and duplicated (dark blue). Fragmented = yellow, missing = red. Percentages for each metric are present in each bar: Csc = complete and single copy, Cd = complete and duplicated, Fr = fragmented, M = missing. For both (A) and (B) “*Orbicella faveolata* (short-read)” is the previously assembled short-read genome, and “*Orbicella faveolata* (long-read)” is the *de-novo* assembly using PacBio HiFi reads

**Fig. 4:**
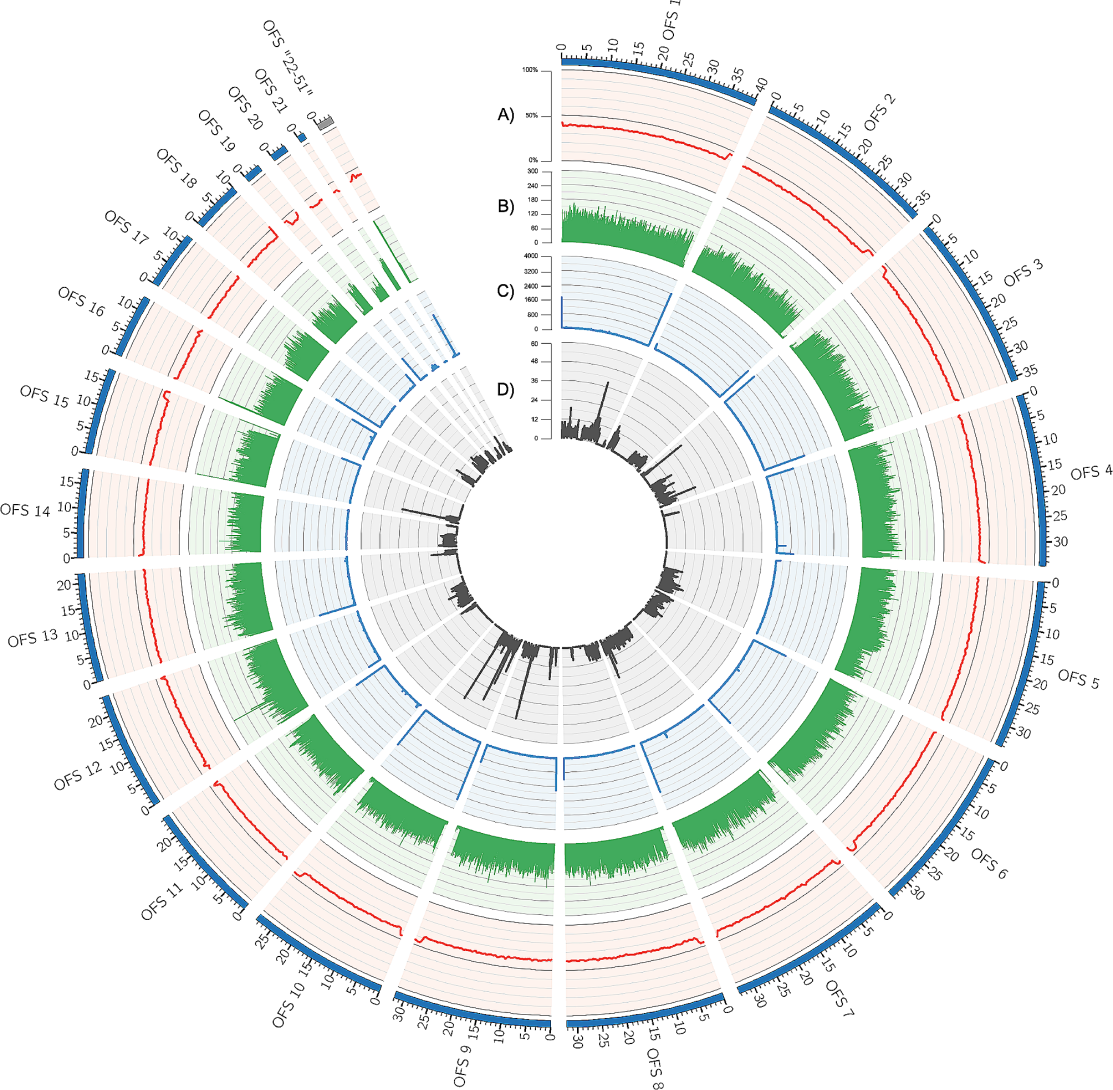
Visualization of scaffolded genome assembly of *Orbicella faveolata*. *A*. GC content calculated with a sliding window of 50,000 base pairs (bp). Y-axis shows the percentage calculated for GC content over each 50,000 bp sliding window. *B*. Repeat content plotted using a sliding window of 50,000 base pairs and the gff output file from RepeatMasker. Y-axis shows counts of repetitive regions for each sliding window of 50,000 base pairs. *C*. Telomeric repeats generated with a sliding window of 50,000 base pairs and the repeat pattern of “TTAGGG”. Y-axis shows the counts of the telomeric repeat for each sliding window of 50,000 base pairs. Telomeric repeats can be identified by peaks at either the start or end of each scaffold. *D*. Gene density calculated with a sliding window of 50,000 base pairs and the “gene” identifiers from the gff file generated from funannotate::annotate. Y-axis shows the counts of genes for each sliding window of 50,000 base pairs

CF HiFi reads were assembled into primary and alternate pseudo-haplotype assemblies with HiFiasm [33]. Kmer profile analysis of primary and alternate assemblies with Merqury [34] confirmed successful duplicate purging (Fig. 2B). The primary assembly consisted of 62 contigs, with a largest contig of 40,246,328 bp, N50 of 33,295,526 bp, L50 of 7, and GC content of 39.49% as identified by Quast [35, 36]. BUSCO [37] analysis of the primary assembly identified 94.86% of metazoan single copy orthologs to be complete (single copy: 93.61%, duplicated: 1.26%), fragmented to be 2.31%, and missing 2.83%. For Quast [35, 36] and BUSCO [37] results of the alternate pseudo-haplotype, please see Supplementary File 1. Scaffolding of the primary assembly with ntLink [38] yielded 51 scaffolds and unchanged quality metrics from the primary assembly (Fig. 3A). BUSCO [37] results were also unchanged as a result of scaffolding (Fig. 3B). Hard and soft masking of scaffolds resulted in 50.20% (247,928,041 bp) of bases masked (Fig. 4). For a full breakdown of masking results, please see Supplementary File 2.

Telomere analysis, using tidk (https://github.com/tolkit/telomeric-identifier), identified 19 scaffolded contigs with telomeric repeats (TeloScafs) at either one (telocentric, 12 of the 19 scaffolds), or both (metacentric, 7 of the 19 scaffolds), ends (Fig. 4). BUSCO [37] analysis of the 19 TeloScafs identified 90.2% metazoan single ortholog copies to be complete (single copy: 89.4%,duplicated: 0.8%), fragmented of 2.4%, and missing of 7.4%.

### Genome annotation

There were 10 RNA-seq samples (Table 1) that were successfully pooled in equal concentration and used for ISO-seq library prep. ISO-seq (https://github.com/PacificBiosciences/IsoSeq) processing of the CCS HiFi reads resulted in 310,755 high quality (hq) transcripts with an average transcript count of 14. Prior to PASA [39] an initial cleaning, using seqClean [40], of the hq transcripts resulted in validation of 310,572 transcripts (1,926 trimmed) and the removal of 183 transcripts (by dust: 10, by short: 173). PASA [39] analysis resulted in 57,279 gene model assemblies. From this, TransDecoder (https://github.com/TransDecoder/TransDecoder) identified 56,835 coding sequences (CDs), with 53,673 open reading frames (ORFs) which could be propagated to the genome. These ORFs were used as input to Funannotate::predict [41] to train ab-initio gene predictors and generate consensus gene models.

**Table 1:** Summary of abiotic and biotic exposure treatments used for fragments of *Orbicella faveolata* to generate as full as possible transcriptional snapshot for annotated transcriptome generation. Ten samples were successfully extracted, clean and concentrated, equally pooled and sent for ISO-seq library preparation and sequencing on one flow cell of a Pacbio Sequel II.

Sample ID	Name	Treatment	Nanodrop 260/280	Nanodrop 260/230	Qubit (ng/ul)
ofav_rna_1	cold stress	Fragment placed at 20ºC for 2 h	2.11	2.01	195
ofav_rna_2	fed	Fragment fed with Reef Roids (PolypLab) for 1 h	2.11	2.09	87.6
ofav_rna_3	heat stress	Fragment placed at 33ºC for 2 h	2.07	1.93	55.3
ofav_rna_4	fighting	Fragment with mesenterial filament extension when next to a different genot of *O. fav*	1.92	1.92	186
ofav_rna_5	night	Fragment sampled at 11pm local time	2.13	1.89	121
ofav_rna_6	day	Fragment sampled at 11am local time	2.13	2.13	117
ofav_rna_7	sperm	Preserved sperm used for HMW DNA	2.17	2.16	35
ofav_rna_8	hypersalinity	Fragment placed in 45ppt for 2 h	2.07	1.96	59.6
ofav_rna_9	disease	Fragment with visible SCTLD lesion	2.09	2	189
ofav_rna_10	hyposalinity	Fragment placed in 20ppt for 2 h	2.11	2.01	63.6

Funannotate::predict [41] identified an initial 32,280 gene models from Evidence Modeler (EVM), which was reduced to 28,663 mRNA gene models after filtering (too short: 5, transposable elements: 3,612). tRNAscan-SE [42] identified 6,033 valid non-overlapping models, resulting in a total of 34,496 (EVM + tRNA-scan) gene models (Supplementary File 3). Refinement of gene models and untranscribed regions (UTRs), using funannotate::update [41], resulted in 1,881 new gene models with this comprising 32,172 protein coding genes, 5,762 tRNAs, and an average gene length of 5977.66 bp (Supplementary File 3). UTRs were also successfully updated for 14,722 gene models. BUSCO [37] analysis of the protein coding genes (database metazoa_odb10) identified complete orthologs of 95.1% (single copy 88.7%, duplicated 6.4%), fragmented as 2.5%, and missing as 2.4% (Supplementary Fig. 1A). For comparison of our protein coding genes to other long-read coral genomes, an Orthofinder [43] analysis was undertaken using the protein fasta files from other publicly available long-read coral genomes [5–10]. Ortholog analysis of the protein coding genes, using Orthofinder [43], identified 29,917 (93%) of genes present in orthogroups, 2,255 (7%) genes not assigned orthogroups, 18,199 (55.7%) genes shared between the coral species s, and 1,903 (5.9%) of genes only present in a single species (Supplementary Fig. 1B, and Supplementary File 4).

### Comparison to previous *O. faveolata* reference genome and other long-read coral genomes

Comparison of BUSCO [37] and Quast [35, 36] metrics demonstrated improvement of our *de-novo* assembly in comparison to the current *O. faveolata* reference assembly (NCBI accession GCA_002042975.1) generated using short-read technology [31]. Scaffold number decreased from 1933 to 51, N50 increased from 4,771,691 bp to 40,246,328 bp, and L50 decreased from 124 to 7 (Fig. 3A). There was also an 8.3% increase of BUSCO [37] completeness (single and duplicated) from 85.3 to 93.6% (Fig. 3B). Alignment of the previous reference genome to our *de-novo* assembly resulted in 99.48% of contigs mapping to our new *de-novo* assembly.

When comparing coding genes, the short-read assembly housed 32,587, while the *de-novo* assembly housed 32,172 (a decrease of 415). Using the protein coding genes, there was also an increase of complete (single copy and duplicated) BUSCOs of 7.9% between the short-read assembly (87.2%) and our *de-novo* genome assembly (95.1%) (Supplementary Fig. 1A).

Comparison of our *de-novo* assembled genome to other coral long-read genomes [5–10] identified comparable BUSCO completeness (single and duplicated) of > 90% (Fig. 3B). Interestingly, Quast [35, 36] identified that our assembly showed lower scaffold count than other long-read assemblies, with the next most contiguous assembly comprising 212 scaffolds compared with the present studies 51 scaffolds (Fig. 3A). When comparing the longest contig and N50 with other publicly available genomes, our *de-novo* assembly was second to the *Montipora capitata* V3 [9] genome resource (Fig. 3A). Comparison of protein coding genes identified comparable BUSCO [37] completion of > 90% between our *de-novo* assembly and other long-read coral genomes (Supplementary Fig. 1A). Once taking into account the total starting number of protein coding genes (Supplementary Fig. 1B), ortholog analysis between the other available long-read coral genomes identified comparable statistics, with > 92% of genes present in all the coral species, and < 7.5% remaining unassigned (Supplementary Fig. 1C). The percentage of orthogroups between species ranged between 50 and 60%, with the percentage of genes in species-specific orthogroups ranging from 2 to 12% (Supplementary Fig. 1C).

### Mitochondrial genome

MitoHiFi [44] identified a complete mitochondrial genome sequence present in the raw HiFi reads with a length of 17,083 bp, successful circulation, and the following genes: two transfer RNAs (tRNAs), 13 protein coding genes, and two ribosomal RNAs (rRNAs). Phylogenetic comparison against all available coral mito genomes on the NCBI identified our *O. faveolata* mitochondrial genome to be placed ‘sister’ to the previous *O. faveolata* mitochondrial genome [45] and other *Orbicella* species (Supplementary Fig. 2). This is most likely due to inherent differences between long and short read sequencing technologies [46, 47].

## Discussion

### Long-read sequencing provides a highly contiguous reference genome resource for *Orbicella faveolata*

In the present study we have demonstrated that long-read PacBio CCS sequencing dramatically improves the genome resource of *Orbicella faveolata*. The previous reference assembly for *O. faveolata* [31] utilized short-read sequencing methodologies on HiSeq 2500 and MiSeq machines, which pose computational challenges for the construction of a highly contiguous assembly [1–4]. Long-read technology, such as PacBio Sequel sequencing, can span repetitive regions of the genome, resulting in fewer contigs. This advantage is clearly demonstrated in our final assembly which consisted of 51 scaffolds, nearly 40 times fewer than the 1,932 scaffolds in the previous short-read *O. faveolata* reference assembly [31]. This increase in contiguity is further reflected in improved N50 (40,246,328 versus 4,771,691), L50 (7 versus 124), and BUSCO completeness (single copy and duplicated, 85.3% versus 93.6%, Fig. 3B) metrics. Despite these improvements, our new *de-novo* assembly identified similarities for GC content (*de-novo*: 39.49%, short-read: 38.5%, Fig. 3A), overall genome length (*de-novo*: 494,730,336 bp, short-read: 485,548,939 bp, Fig. 3A) with the short-read *O. faveolata* genome resource [31], as well as a ploidy of two (Fig. 2A). Comparison between protein coding genes also identified an improvement between our *de-novo* assembly and the previous *O. faveolata* reference, with this identified through an increase of BUSCO completeness (single copy and duplicated) from 87.2 to 95.1% (Supplementary Fig. 1B). These results highlight how long-read methodologies can improve upon older genomic resources that used short-read methodologies.

We also compared our assembly to other publicly available long-read stony coral genome assemblies [5–10]. Despite only using HiFi reads for our assembly and scaffolding, our assembly attains approximately equal completeness and contiguity as measured by Quast [35, 36] (Fig. 3A) and BUSCO [37] (Fig. 3B) when compared to assemblies that incorporated auxiliary scaffolding techniques [7, 9]. With continued improvement and cost reduction of long-read sequencing methodologies, the results of our study show that the generation of a high quality reference genome for stony corals can be achieved without additional methods such as Hi-C, optical mapping, or supplemental short-read sequencing. Using these additional methods are still advantageous, allowing additional decreases in contig number, as well as generation of chromosomal level assemblies. BUSCO completion (single copy and duplicated) of the protein coding genes were also comparable between our *de-novo* assembly and the other coral long-read genomes, indicating it is of comparable quality despite only using HiFi reads (Supplementary Fig. 1A). Orthofinder [43] analysis identified 93% of protein coding genes from our *O. faveolata de-novo* assembly to be assigned to orthogroups when analyzed with other long-read coral genome resources [5–10], with 5.9% of these genes being species specific to *O. faveolata* (Supplementary 4). This suggests our gene prediction and annotation pipeline is of comparable quality to other coral long-read genome assemblies. As more long-read coral genomes become available, an in depth analysis of orthologs and paralogs should be undertaken to identify core coral gene function, and potential processes which could be species specific.

### Potential chromosomes are recovered from HiFi reads without additional sequencing information

Previous work has suggested that the potential karyotype of *O. faveolata* is 16 chromosomes [48]. In our study, telomeric repeat analysis identified regions at either one (telocentric, 12 scaffolds) or both (metacentric, seven scaffolds) ends of 19 of the 51 scaffolds (TeloScafs) (Fig. 4). Telomeric repeats are indicative of chromosome ends, suggesting several scaffolds in our assembly represent complete, telomere-to-telomere sequences, and thus that we may have captured some full chromosomes in our assembly. BUSCO [37] analysis also identified 90.2% of metazoan universal single copy orthologs as complete in telomere containing scaffolds, as compared to 94.86% in the entire scaffold set (Fig. 3A). With the high percentage BUSCO completion of the telomere containing scaffolds, this further suggests that several scaffolds likely represent complete chromosomes. The number of identified TeloScafs is however larger than the potential karyotype of 16 for *O. faveolata* [48] suggesting that we missed some repetitive sections of the genome such as centromeric repeats. This may also be due to only utilizing HiFi reads for our assembly. Future work should re-assemble our HiFi reads using additional methodologies such as optical mapping [49, 50] or Hi-C sequencing [51, 52] to achieve a true telomere-to-telomere chromosome scale assembly and resolve any discrepancies in the number of telomere-containing scaffolds. Additionally, the karyotype of *O. faveolata*, as well as other coral species, should be defined experimentally rather than relying on bioinformatic methods to infer karyotype. Historically, working with coral gametes has been difficult due to them only spawning once a year. With developments in ex-situ spawning, there is now higher availability of coral gametes throughout the year [53–55] making gamete based research easier and more accessible for coral species. This, paired with new karyotyping methodologies for non-model invertebrate organisms [56], will allow experimental identification of coral species karyotype to occur, paving the way for improved genome assemblies due to known chromosome number.

### Future directions and conclusions

In this study we provide an updated genome resource for the endangered coral species *O. faveolata* at near-chromosome scale using only PacBio HiFi long reads. Despite improvements in completeness and contiguity over the current *O. faveolata* reference assembly [31], our assembly may yet be improved to a bonafide chromosomal level with additional sequencing (specifically Hi-C). Use of this updated resource will also assist efforts to functionally characterize genes, an area of research that is just starting to occur within coral species [57]. Additionally, we hope that this resource will facilitate more in-depth ‘omic analyses utilizing *O. faveolata* as the focal species. As this species continues to be integrated into reef restoration activities [19–21], a thorough understanding of its population structure and response to anthropogenic stressors will be key to its preservation.

## Methods

### Tissue collection, nucleic acid extractions, library preparation, and sequencing

To generate high molecular weight (HMW) DNA for de-novo genome assembly, gamete bundles (sperm and eggs) were collected from one spawning colony of *Orbicella faveolata* (Fig. 1A) on the 18th August 2022 at roughly 00:15 local time at Horseshoe Reef (Key Largo, FL, USA; 25.1388°N, 80.2950°W). Gamete bundles were collected in a conical mesh net with a 50 ml conical centrifuge tube at the apex (Fig. 1B), then capped and brought to the boat. Onboard the vessel, as gamete bundles started to break apart, they were diluted with filtered seawater to reach a sperm concentration of ~ 10^8^ cells/ml [58]. After transport to the University of Miami Rosenstiel School, eggs were separated from sperm using a Corning 70 μm sterile cell strainer. Eggs caught on the filter were discarded, and filtrate was inspected under a microscope to remove any residual eggs. Six 1.5 ml tubes with 1 ml of the sperm filtrate, in seawater, were then centrifuged at 3,000 g for five minutes. The supernatant was removed, and 1 ml of additional sperm filtrate was added to each tube and repeated 8x. Each tube had a total of 8 ml of filtrate processed. Pelleted sperm was then resuspended in 1x PBS (pH 7.2) using a wide pipette tip, and centrifuged at 3,000 g for five minutes. The supernatant was removed without disturbing the pellet, and each tube was then flash frozen in liquid nitrogen and stored at -80ºC. Frozen sperm was then shipped to the University of California (UC) Davis Genome Center for HMW DNA extractions, library preparation, and sequencing on one flow cell of a PacBio Sequel II. For detailed methods, please see Supplementary File 5.

To generate a high quality and complete annotated transcriptome, the largest transcriptional snapshot of mRNA was desired to capture all transcripts that are present within the *O. faveolata* genome. As such, we exposed fragments (~ 5 cm^2^) from one genet of *O. faveolata* to different biotic and abiotic stimuli to maximize the range of mRNA expression (Table 1). This *O. faveolata* genet was a rescue coral that had been housed in the Experimental Reef Lab (Miami, FL) for three months prior to use in the biotic and abiotic exposures for RNA expression profiles. Following stimuli exposure, coral fragments were sampled using a hammer and chisel and placed in a 2 ml bead beating tube filled with 0.1 and 0.5 mm beads, and 1.2 ml of DNA/RNA shield (Zymo, Irvine). Bead beating tubes were then bead beat for 30 min on a VortexGenie at max speed before being centrifuged at 16,000 rpm for 1 min. A total of 400 µl of supernatant was transferred to a new tube and total RNA was extracted with the Quick RNA Miniprep kit (Zymo, Irvine) including the fifteen minute DNase *I* digestion step. Total RNA was eluted with 80 µL of pre-heated (60 ^o^ C) RNase-free water, with a three minute incubation on the spin column matrix. Eluted total RNA was cleaned and concentrated with the Clean and Concentrate − 5 RNA kit (Zymo, Irvine), with an elution volume of 25 µl of pre-heated (60 ^o^C) RNase-free water. The purity and concentration of the RNA was assessed using a Nanodrop and a Qubit V4 (Invitrogen), respectively. RNA was then sent to UC Davis DNA Technology Core (Davis CA) for additional QC (TapeStation), library prep, and sequencing on one flow cell of a PacBio Sequel II. For detailed methods, please see Supplementary File 5.

### Mitochondrial genome assembly

The mitochondrial genome was assembled from the HiFi reads, prior to contaminant removal, using MitoHiFi [44, 59] with key parameters -o 5 (invertebrate parameter). Due to unsuccessful circulation using the publicly available *O. faveolata* mitochondrial genome [45], MitoHiFi was run using closely related stony coral species mitochondrial genomes available on the NCBI (Supplementary File 5). For our final mitochondrial genome assembly, we used *Platygrya carnosa* (Nucleotide accession = NC_020049.1) [60] as the reference in MitoHiFi which allowed successful circulation. Phylogenetic analysis was undertaken with our *O. faveolata* mitochondrial genome and all available Scleractinia coral mitochondrial genomes on the NCBI (Supplementary File 6). Briefly, all reference genomes were concatenated into one fasta and run through trimal [61] with the following parameters: -gt 0.3, -st 0.001, -cons 30. Circulator [62] was used to orient all mitochondrial genomes in the same order, before multi-sequence alignment with mafft [63]. RAxML [64] was then used to generate the phylogenetic tree (-x 10, -p 10, -#100, -m GTRCAT) with the default value of 100 bootstraps. The phylogenetic tree was visualized with figtree (http://tree.bio.ed.ac.uk/software/figtree/), ggplot [65] and ggtree [66] in R [67] and RStudio.

### *De-novo* genome assembly

A schematic of the bioinformatic pipeline used for *de-novo* genome assembly can be found in Supplementary Fig. 3. Raw HiFi reads first underwent a contamination screening, following the methodology in [68], using BLASTn [32, 68] against the assembled mitochondrial *O. faveolata* genome and the following databases: common eukaryote contaminant sequences (ftp.ncbi.nlm.nih.gov/pub/kitts/contam_in_euks.fa.gz), NCBI viral (ref_viruses_rep_genomes) and prokaryote (ref_prok_rep_genomes) representative genome sets downloaded with blast::update_blastdb.pl. All raw HiFi reads with a bit score > 1000 were removed. Prior to assembly, the kmer profile of cleaned raw HiFi reads was generated with Meryl [34], and used for genome profiling with GenomeScope2 [69] to estimate genome size, repetitiveness, heterozygosity, and ploidy. The cleaned raw HiFi reads were then assembled with HiFiasm [33] (key parameters:–primary, -s 0.55,–purge-max 150) into a primary and alternative assembly. Assembly statistics were obtained using Quast [35, 36], BUSCO [37] (organism metazoa_odb10), and Merqury [34]. A subsequent BLASTn [32] was run to identify additional contaminants using the previously mentioned databases. Scaffolding of the primary assembly was done using the clean raw HiFi reads and nt-links (key parameters: g 100, rounds 5) [38, 70] resulting in the scaffolded assembly. Final assembly statistics were generated with BUSCO [37] and Quast [36].

The scaffolded assembly was then analyzed with RepeatModeler2 [71] to generate a *de-novo* library of repetitive elements. RepeatModeler2 [71] results were uploaded to the Dfam database (https://www.dfam.org/home) as requested in the user documentation. Output from RepeatModeler2 [71] was then used in RepeatMasker (https://github.com/rmhubley/RepeatMasker) to generate hard masked (default parameters) and soft masked (-xsmall) versions of the scaffolded assembly with accompanying gff files.

### Identification of telomeric repeats in the scaffolded contigs

To identify potential telomeres in our scaffolded contigs (TeloScafs), the Telomere Identification Toolkit (tidk; https://github.com/tolkit/telomeric-identifier) with the coral telomeric repeat “TTAGGG” [72, 73] was used with following parameters: search,–window 50,000. Scaffolded contigs with telomeric repeats at either one (telocentric) or both (metacentric) ends were then used in a BUSCO [37] (database = metazoa_odb10) analysis as to allow comparison of BUSCO completeness between the set of TeloScafs, and the scaffolded de-novo assembly.

### Annotation of *de-novo* genome and transcriptome assemblies

A combination of PASA [39] and funannotate [41] were used to annotate the *de-novo* assembled genome. PASA [39] was used to model gene structures using the scaffolded genome assembly and the high quality (hq) transcripts. The hq transcripts were cleaned using seqClean [40] before being used for transcript alignment and alignment assembly with the scaffolded genome assembly (pasa::Launch_PASA_pipeline.py, key parameters: -R, -T,–ALIGNERS blat,minimap2,–TRANSDECODER,–ALT_SPLICE). A high quality dataset for downstream ab initio gene prediction containing gene models with coordinates based on the genome sequences was generated from PASA transcript assemblies with pasa::pasa_asmbls_to_training_set.dbi. The soft masked scaffolded genome and transcript based gene models from PASA [39] were then input into funannotate::predict [41] (key parameters:--organism other,–repeats2evm,–keep_evm,–optimize_augustus) to train the *ab-initio* gene predictors (Augustus [74], GeneMark-ES/ET [75], snap [76], glimmerhmm [77]), before running Evidence Modeler [78, 79] to generate consensus gene models. Transfer RNA’s (tRNAs) were identified using tRNAscan-SE [42]. Gene model predictions and untranscribed regions (UTRs) were then refined using funannotate::update [41] using the hq transcripts and a *de-novo* assembled transcriptome of *O.faveolata* (from [80]) using Trinity [81] with key parameter:–trimmomatic. InterproScan [82] was then run on the updated gene models to classify proteins into families, and predict domains. Interproscan [82] results were then incorporated with the results from funannotate::update [41] and used in funannotate::annotate [41] to assign functional annotation to the protein-coding genes, with the optional addition of eggNOG-mapper [83–85].

### Comparisons to other coral genome resources

To compare our final *de-novo* assembly to the previous *Orbicella faveolata* reference genome [31] BUSCO [37] (database = metazoa_odb10) and QUAST [35, 36] were used. Percentage mapping of reads between the two genomes was done using Minimap2 [86, 87] (key parameters: -ax asm5) and samtools [88] (key parameter: flagstat). Comparison of coding genes was done using the protein fasta files in BUSCO [37] using the proteins flag (-m) and database metazo_odb10.

Our final *de-novo* assembly was compared against all other publicly available long-read coral genomes [5–10] using QUAST [35, 36] and BUSCO [37] (database = metazoa_odb10). An additional analysis of coding genes was run using the protein fasta files from the long-read assemblies in BUSCO [37] with key parameters -m protein and database metazo_odb10. Finally, Orthofinder [43] was used to identify ortholog groups between all the long-read coral genomes with results visualized using ggplot2 [65].

### Summary circos plot generation

Circos [89] was used to generate a circular summary figure of the *de-novo* assembled genome. For visualization, all contigs less than 1 mb were combined. Additional quality metrics were calculated as follows, with outputs formatted for Circos using tidyverse [90] and SeqinR [91] in Rstudio. GC content and skew were identified using GCcalc (https://github.com/WenchaoLin/GCcalc) with key parameters: -w 50,000, and -s 250,000. For repeat content, the GFF from repeatmasker (https://github.com/rmhubley/RepeatMasker) was first converted to a bed file using Bedops [92] before being used in deepStats::dsComputerBEDdensity [93] with a sliding window of 50,000 (-w 50,000). For gene content, the GFF file from funannotate::update [41] was processed in the same manner as repeat content above. The output from the telomere analysis, using tidk (https://github.com/tolkit/telomeric-identifier), was also incorporated in the final Circos [89] summary figure.

### Electronic supplementary material

Below is the link to the electronic supplementary material.

Supplementary Material 1

Supplementary Material 2

Supplementary Material 3

Supplementary Material 4

Supplementary Material 5

Supplementary Material 6

Supplementary Material 7

Supplementary Material 8

Supplementary Material 9

Supplementary Material 10

## Data Availability

The *O. faveolata de-novo* genome assembly presented here is publicly available at PRJNA970355 on the NCBI, as well as at 10.5281/zenodo.10151798. Full analysis scripts, pipeline, and tool versions are avaliable at https://github.com/benyoung93/orbicella_faveolata_pacbio_genome_transcriptome. All program versions are available in Supplementary File 7 and in the github repository. The mitochondrial genome is available at GenBank accession OR906199, as well as at 10.5281/zenodo.10151798.
