# Supplementary material for: Annotated genome and transcriptome of the endangered Caribbean mountainous star coral (Orbicella faveolata) using PacBio long-read sequencing
Source: BMC Genomics. 2024 Feb 29;25:226. doi: 10.1186/s12864-024-10092-w (PMC10905781; doi:10.1186/s12864-024-10092-w)
Supplement: Supplementary file 3 — Supplementary Material 3 [file 12864_2024_10092_MOESM3_ESM.docx]

## HMW DNA extraction, library prep and sequencing

HMW DNA was extracted from flash frozen sperm. 1.5ml of lysis buffer (100 mM NaCl, 10 mM Tris-HCl pH 8.0, 25 mM EDTA, 0.5% (w/v) SDS) and 100µg/ml Proteinase K were added to the sperm sample, and incubated at room temperature for two hours until the solution was homogenous. The lysate was treated with 20µg/ml RNase A at 37^0^C for 30 minutes, and cleaned with equal volume of phenol/chloroform using phase lock gels (Quantabio Cat # 2302830). DNA was precipitated by adding 0.4X volume of 5M ammonium acetate and 3X volume of ice cold ethanol. The DNA pellet was then washed twice with 70% ethanol and resuspended in elution buffer (10 mM Tris, pH 8.0). Purity of gDNA was assessed using a NanoDrop ND-1000 (observed 260/280 ratio of 1.82 and 260/230 ratio of 2.41), concentration using a Qubit 2.0 fluorometer (observed total yield of 15µg) and integrity using Femto pulse system (observed majority of DNA >100kb). Library preparation followed in the SMRTBell Prep kit v3.0 with no deviations from manufacturer's protocol. Briefly, HMW gDNA was sheared to target DNA size distributions between 15-18 kb using Diagenode’s Megaruptor 3 system. Sheared gDNA was concentrated using 1x of SMRTbell cleanup beads for the repair and A-tailing incubation (37 ºC for 30 minutes, and 65 ºC for 5 minutes), followed by ligation of overhang adapters (20 ºC for 30 minutes), clean-up using 1x SMRTbell cleanup beads, and nuclease treatment (37 ºC for 15 minutes). The SMRTbell library was size-selected (3.1x of 35% v/v diluted AMpure PB beads) to progressively remove SMRTbell templates <5 kb. The 15-18 kb average high fidelity (HiFi) SMRTbell library was then sequenced using one 8M SMRT cell (Pacific Biosciences, Menlo Park, CA; Cat# 101-389-001), Sequel II sequencing chemistry 2.0, and 30-hour movies on a PacBio Sequel II sequencer. HiFi reads (predicted accuracy> = Q20) were then generated from the sequencing run, using CCS (ref), for downstream *de-novo* genome assembly.

## ISO-seq QC, library prep, sequencing, and initial processing

Complementary DNA (cDNA) was synthesized from pooled total RNA using the NEBnext Single Cell/Low Input cDNA synthesis and Amplification Module with 15 cycles of PCR used during amplification. cDNA was purified using 0.86x SMRTbell cleanup beads before SMRTbell library preparation was done using the SMRTbell prep kit 3.0. The ISO-seq SMRTbell prep library was sequenced using one 8M SMRT cell, Sequel II sequencing chemistry 2.0, and 24-hour movies on a PacBio Sequel II sequencer. The sequencing run was first processed using CCS to generate HiFi reads, before following the ISO-seq::CLI workflow (<https://isoseq.how/umi/cli-workflow.html>) to generate polished isoforms. For all downstream steps, the high quality (hq) transcripts (predicted accuracy >0.99) were used.
