# Supplementary material for: Annotated genome and transcriptome of the endangered Caribbean mountainous star coral (Orbicella faveolata) using PacBio long-read sequencing
Source: BMC Genomics. 2024 Feb 29;25:226. doi: 10.1186/s12864-024-10092-w (PMC10905781; doi:10.1186/s12864-024-10092-w)
Supplement: Supplementary file 8 — Supplementary Material 8 [file 12864_2024_10092_MOESM8_ESM.docx]

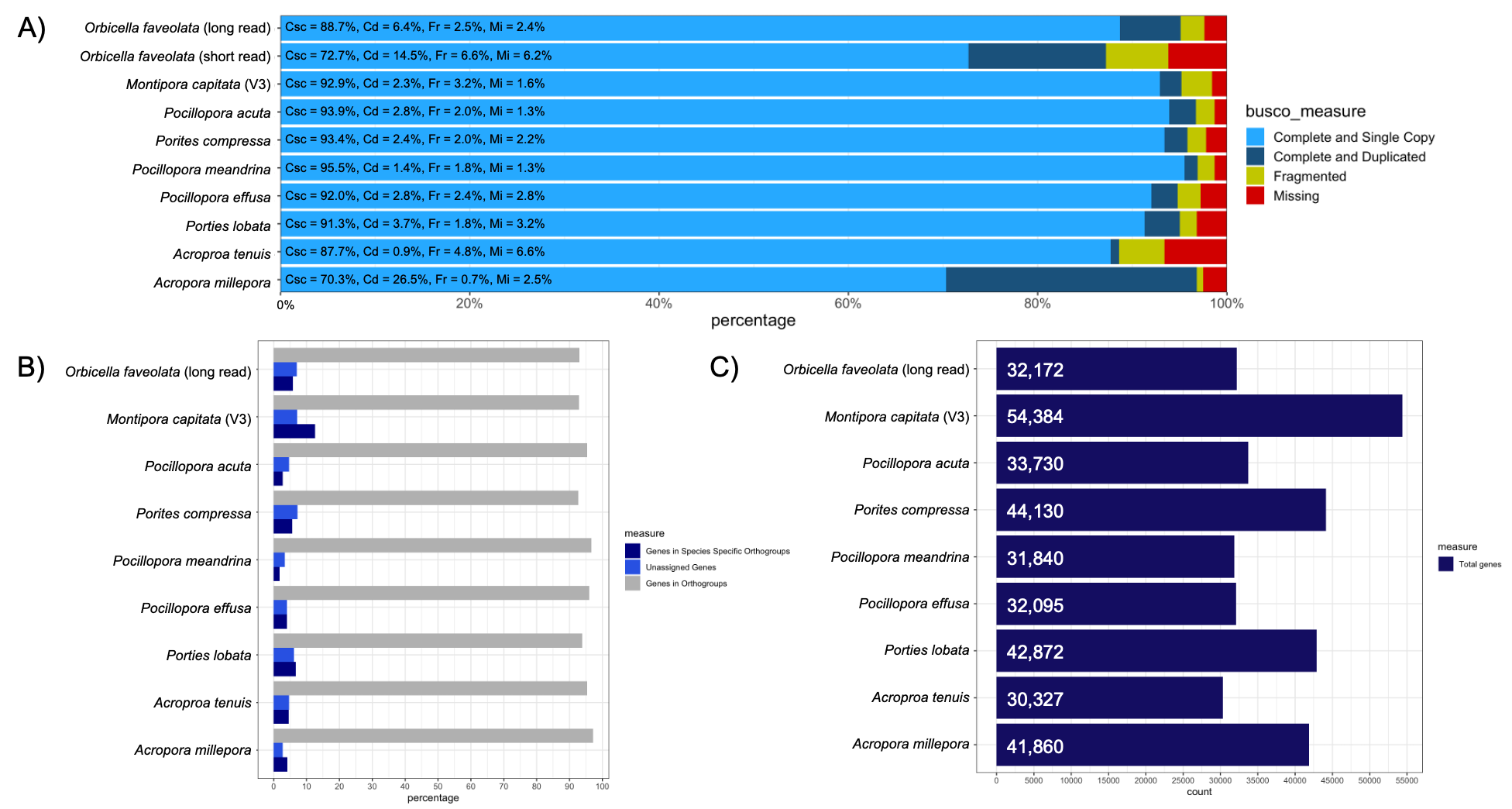


**Supplementary Figure 1 - Results from the analysis of BUSCO and Orthofinder on the protein coding genes from our *de-novo* assembly, previous *O. faveolata* reference genome, and other long-read coral genomes.**

1. Results of BUSCO (database = metazoa_odb10) analysis on the protein coding genes on our *de-novo* assembly, the previous *O. faveolata* reference genome, and other long read coral genomes. Completeness is split into single copy (light blue) and duplicated (dark blue). Fragmented = yellow, Missing = red. Percentages for each metric are present in each bar: Csc = complete and single copy, Cd = complete and duplicated, Fr = fragmented, M = missing. “*Orbicella faveolata* (short read)” is the previously assembled short-read genome, and “*Orbicella faveolata* (long read)” is our *de-novo* assembly using PacBio HiFi reads.
2. Results from Orthofinder analysis between our *de-novo* assembly and other publicly available coral long-read genomes
3. Total number of protein coding genes present in coral long-read genomes used in Orthofinder analysis. Number within the bar shows the total number of protein coding genes present in each long-read genome assembly.
