# Supplementary material for: Annotated genome and transcriptome of the endangered Caribbean mountainous star coral (Orbicella faveolata) using PacBio long-read sequencing
Source: BMC Genomics. 2024 Feb 29;25:226. doi: 10.1186/s12864-024-10092-w (PMC10905781; doi:10.1186/s12864-024-10092-w)
Supplement: Supplementary file 9 — Supplementary Material 9 [file 12864_2024_10092_MOESM9_ESM.docx]

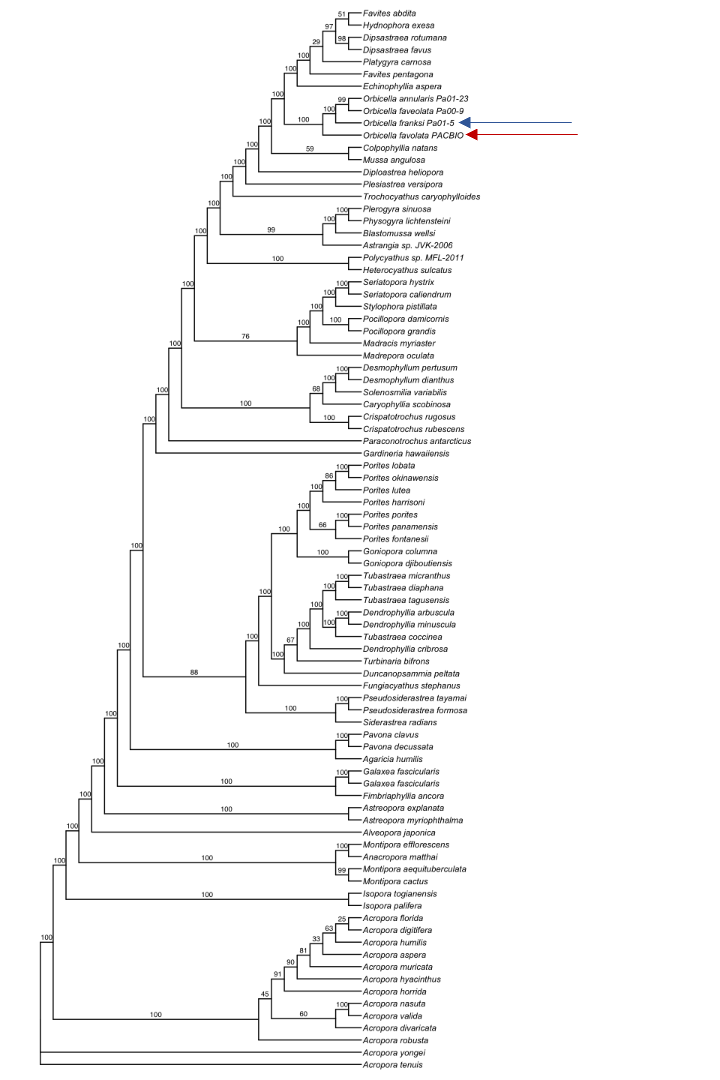


**Supplementary Figure 2 - Phylogenetic placement and gene content of the new *Orbicella faveolata* mitochondrial genome.**

Phylogenetic tree of all available stony coral mitochondrial genomes available on the NCBI. Blue arrow = previous short-read reference mitochondrial genome of *O. faveolata*. Red arrow = our *de-novo* assembled mitochondrial genome from *O. faveolata*.
