# Supplementary material for: Annotated genome and transcriptome of the endangered Caribbean mountainous star coral (Orbicella faveolata) using PacBio long-read sequencing
Source: BMC Genomics. 2024 Feb 29;25:226. doi: 10.1186/s12864-024-10092-w (PMC10905781; doi:10.1186/s12864-024-10092-w)
Supplement: Supplementary file 10 — Supplementary Material 10 [file 12864_2024_10092_MOESM10_ESM.docx]

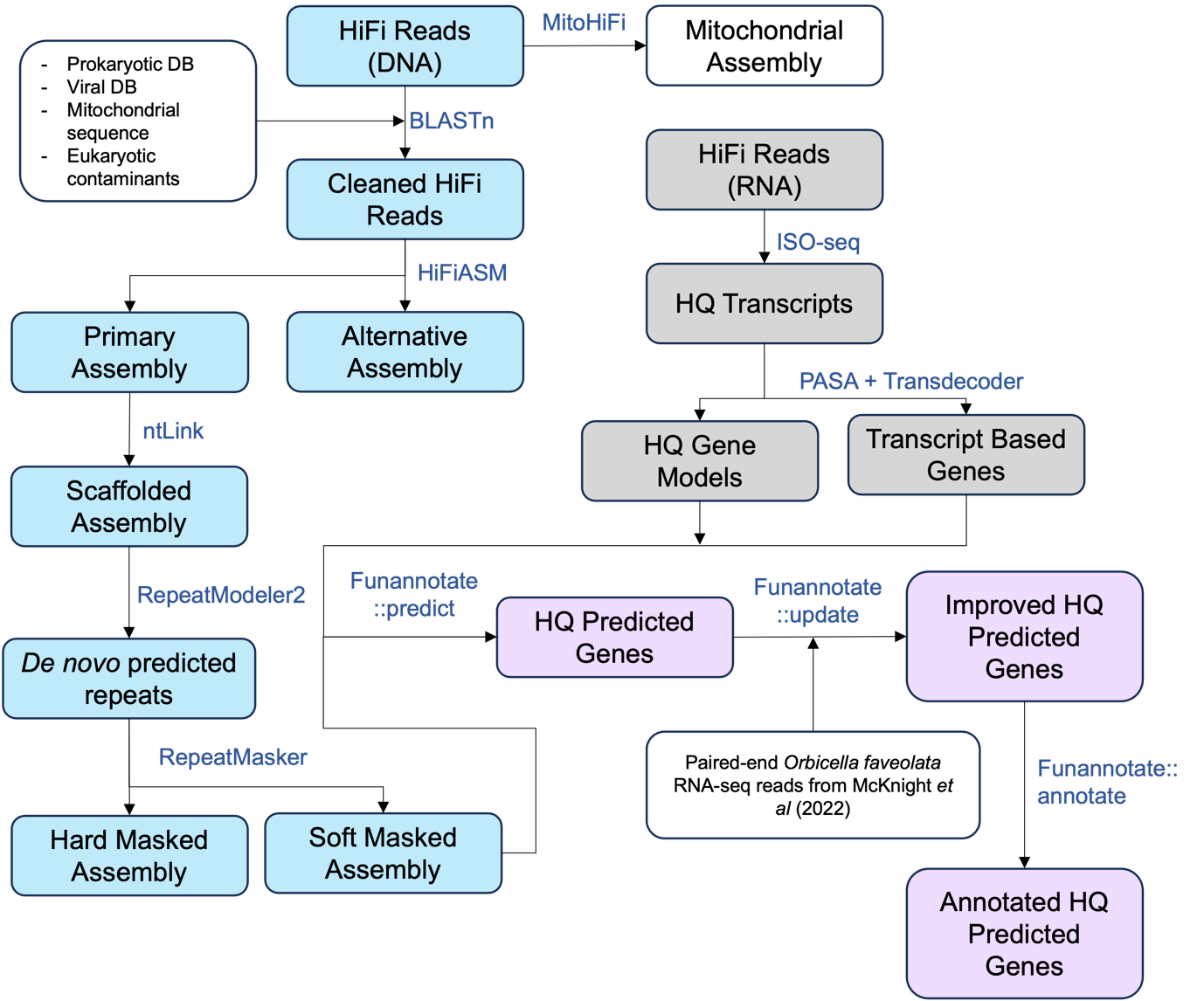


**Supplementary Figure 3 - Bioinformatic pipeline for *de-novo* genome and transcriptome assembly**

Full bioinformatic pipeline for genome (blue boxes), transcriptome (gray boxes), and their annotation (purple boxes). Blue filled boxes show raw DNA HiFi reads through to soft and hard masked *de-novo* genome assemblies. Grey filled boxes show RNA HiFi reads used to generate high quality gene models and transcript-based genes. Purple filled boxes show gene prediction, updating, and annotation using outputs from the blue (DNA) and gray (RNA) processing pipelines. White fill boxes show additional input/output datasets used/generated during the bioinformatic pipeline.
